# Supplementary material for: Systems Approach to Investigate the Role of Fruit and Vegetable Types on Vascular Function in Pre-Hypertensive Participants: Protocol and Baseline Characteristics of a Randomised Crossover Dietary Intervention
Source: Nutrients. 2024 Sep 1;16(17):2923. doi: 10.3390/nu16172923 (PMC11397325; doi:10.3390/nu16172923)
Supplement: Supplementary file 1 [file nutrients-16-02923-s001.zip › nutrients-3137884-supplementary.pdf]

nutrients

MDPI

Supplemental Materials

Systems Approach to Investigate the Role of Fruit and Vegetable Types on Vascular Function in Pre-Hypertensive Participants: Protocol and Baseline Characteristics of a Randomised Crossover Dietary Intervention

Linda M. Oude Griep [1,2,*], Gary Frost [3], Elaine Holmes [3,4], Nicholas J. Wareham [1] and Paul Elliott [2]

* Correspondence: linda.oudegriep@mrc-epid.cam.ac.uk

Table S1. Participant timeline and measurements performed from initial screening and per clinic visit according to SPIRIT[49].

		Health Screening	Intervention Period 1		Intervention Period 2		Intervention Period 3	
		Visit 0	Visit 1	Visit 2	Visit 3	Visit 4	Visit 5	Visit 6
**Questionnaires**								
Pre-screening questionnaire	Initial eligibility check	X						
Habitual dietary intake	Dietary history interview	X						
	3-day food diary	X						
Screening questionnaire	Demographics, medical history, medication and supplement use, lifestyle		X	X	X	X	X	X
Intervention lifestyle and health questionnaire	General health, self-reported physical activity (IPAQ long form), sleep (9-item PSQI), appetite profiles, quality of life (AQoL-8D), mental well-being (7-item Warwick-Edinburgh Scale), positive and negative affect schedule (PANAS)		X	X	X	X	X	X
Cognitive tests	Paired associate learning, digit span, two choice reaction time, Stroop, numerical and verbal reasoning, vocabulary		X	X	X	X	X	X
**Physical measurements**								
Anthropometrics	Height, weight, waist and hip circumference	X	X	X	X	X	X	X
Body composition	Bioelectrical impedance analysis	X	X	X	X	X	X	X
Blood pressure	Oscillometry (Omron HEM-705 CP)	X	X	X	X	X	X	X
	24-hr ABPM (Mobil-O-Graph NG) [1]		X	X	X	X	X	X
Arterial stiffness	Pulse Wave Velocity (Vicorder)		X	X	X	X	X	X
Physical activity	Accelerometer (AX3)[1]		X	X	X	X	X	X
**Sample collections**								
24-hr urine and spot urine collection [2]	Urinary metabolites including markers of fruit and vegetable intake		X	X	X	X	X	X
Faeces collection[2]	Faecal microbiota composition and analysis		X	X	X	X	X	X
Fasting blood sample	Markers of cardiovascular disease (e.g. lipid profile), inflammation (e.g. CRP), circulation metabolites		X	X	X	X	X	X

	including markers of fruit and vegetable intake

[1] In a subgroup of the study population; [2] 24-hr urine and faecal samples were collected in the 24 hours prior to the clinic visit.

Table S2. Types and estimated weekly and daily portions of fruits and vegetables provided weekly during the control and intervention periods.

	Control Diet		High Fruit and Vegetable Diet		Citrus fruit and Cruciferous Vegetables Diet	
	Types	Estimated portions [1]	Types	Estimated portions [1]	Types	Estimated portions[1]
Fruit	Apple	2	Apple	7	Clementine, 3 packs of 6, 18 total	9
	Banana	3	Banana	8	Grapefruit, 2	4
	Grape, 170 g	2	Grape, 750 g	8.8	Lemon, 5 to sprinkle over food	2
			Pear	4	Orange	5
					Orange juice, 100%, 1L	6.7
Vegetables	Carrot	2	Carrot	6	Broccoli, 1 head of 335 g	4.2
	Sweet pepper, red	2	Carrot batons, 400 g	5	Broccoli & cauliflower florets, 240 g	3
	Tomato	3	Sweet pepper, mixed	3	Brussels sprouts, 500 g	5
			Tomato	7	Cauliflower, 1 head of 400 g	5
			Tomato, cherry, 335 g	4.2	Kale, 200 g	2.5
					Savoy cabbage, 500 g	6.3
Estimated number of portions						
	Per week	14		53		53
	Per day	2.0		7.6		7.5

[1] Portion sizes were allocated according to the Food Standards Agency 3rd Edition 'Food Portion Sizes.
